# Deoxynivalenol triggers the expression of IL-8-related signaling cascades and decreases protein biosynthesis in primary monocyte-derived cells

**DOI:** 10.1007/s12550-024-00528-3

**Published:** 2024-03-18

**Authors:** Constanze Nossol, P. Landgraf, M. Oster, S. Kahlert, A. Barta-Böszörmenyi, J. Kluess, K. Wimmers, B. Isermann, O. Stork, D. C. Dieterich, S. Dänicke, H.-J. Rothkötter

**Affiliations:** 1 Institute of Anatomy, Medical Faculty, Otto-von-Guericke University Magdeburg, Leipziger Strasse 44, 39120 Magdeburg, Germany; 2 Institute for Pharmacology and Toxicology, Medical Faculty, Otto-von-Guericke University Magdeburg, Leipziger Straße 44, 39120 Magdeburg, Germany; 3 Research Institute for Farm Animal Biology (FBN), Wilhelm-Stahl-Allee 2, 18196 Dummerstorf, Germany; 4 Institute of Animal Nutrition, Friedrich-Loeffler-Institute (FLI), Federal Research Institute for Animal Health, Bundesallee 50, 38116 Braunschweig, Germany; 5 Institute of Laboratory Medicine, Clinical Chemistry and Molecular Diagnostics, Medical Faculty, Paul-List-Str. 13-15, 04103 Leipzig, Germany; 6 Institute of Biology, Faculty of Natural Science, Otto-von-Guericke University Magdeburg, Leipziger Strasse 44, 39120 Magdeburg, Germany

**Keywords:** DON, Deoxynivalenol, IL-8, Primary MoDCs, Microarray, AHA, Protein synthesis

## Abstract

Humans and their immune system are confronted with mold-contaminated food and/or mold-contaminated air in daily life and indoor activities. This results in metabolic stress and unspecific disease symptoms. Other studies provided evidence that exposure to mold is associated with the etiology of allergies. Deoxynivalenol (DON) is of great concern due to its frequent occurrence in toxically relevant concentrations. The exposure to this toxin is a permanent health risk for both humans and farm animals because DON cannot be significantly removed during standard milling and processing procedures. However, the direct effect on immunity or hematology is poorly defined because most investigations could not separate the effect of DON-contaminated feed intake. Due to the widespread distribution of DON after rapid absorption, it is not surprising that DON is known to affect the immune system. The immune system of the organism has one important function, to defend against the invasion of unknown substances/organisms. This study shows for the first time a synergistic effect of both—low physiological DON-doses in combination with low LPS-doses with the focus on the IL-8 expression on protein and RNA level. Both doses were found in vivo. IL-8 together with other anorectic cytokines like IL-1β can affect the food intake and anorexia. We could also show that a calcium-response is not involved in the increased IL-8 production after acute DON stimulation with high or low concentrations.

## Introduction

Deoxynivalenol (DON) is due to its frequent occurrence in toxically relevant concentration of high importance with the focus of immune regulation (Bottalico and Perrone 2002; Logrieco et al. 2002). The accumulation of this toxin depends on environmental factors like temperature and humidity and cannot be avoided completely. Therefore, the exposure to this toxin is a permanent health risk for both human and farm animals (Lauren and Smith 2001). Pigs are particularly susceptible to DON and show poisoning symptoms after DON-intake like feed refusal, increased salivation, and vomiting (Rotter et al. 1996). The intoxication due to high DON concentrations is rarely observed. More important is the chronic exposure to lower concentrations of DON, which cause economical losses in animal production due to reduced feed intake and live weight gain. The effects of DON in combination with lipopolysaccharide (LPS) load on the porcine health result from numerous interactions between genetic and physiological factors including immune status, food intake, or microbiome composition. Due to this complex network in animals, it is very difficult to figure out clear cause-effect relations. Prelusky and co-worker showed that the intragastrical application of radio-labelled pure DON results in a rapid absorption, a widespread distribution within the body, and a slower renal elimination as well as a poor phase I/II metabolism in pigs compared to that in more resistant ruminants (Prelusky et al. 1988). Due to the widespread distribution of DON after rapid absorption, it is not surprising that DON is known to affect the immune system. Dendritic cells (DC) as professional antigen-presenting cells are primarily responsible for initiating or suppressing immune response (Bilsborough and Viney 2004; Fleeton et al. 2004). Dendritic cells are located in the lamina propria mucosae, where they are likely to come in contact with DON dietary (Haverson et al. 2000; Mowat 2003). DCs can effect T cell development by modulating the expression of co-stimulatory molecules, cytokines, and chemokines (de Jong et al. 2005). Furthermore, dendritic cells influence the innate immune system by secreting IL-8, because it has a chemotactic effect on neutrophil granulocytes (Matsushima and Oppenheim 1989; Larsen et al. 1989). But first of all, DCs have to be activated, for example, by LPS. Upon activation, DCs need to take up and process molecules from their environment (Banchereau and Steinman 1998), and in the next step, they produce co-stimulatory or co-inhibitory cytokines (Sinclair and Anderson 1996). This study shows a synergistic effect of practical relevant DON and LPS doses with the focus on the IL-8 expression on protein and RNA levels. Different studies have already shown the influence of DON on ribosomes and translation but also the synergistic effect of both (Kullik et al. 2013; Zhou et al. 2014). However, here we have quantified this effect with the BONCAT method which allows a better estimation of the relevance of the ribotoxic impact. We could also show that a calcium response is not involved in the increased IL-8 production after acute DON stimulation.

## Material and methods

### Isolation and generation of monocyte-derived dendritic cells (MoDCs)

Blood samples were taken from two minipigs and samples were processed separately. The monocyte-derived dendritic cells were isolated and cultured as described in Bimczok et al. (2007). The PBMCs (peripheral blood mononuclear cells) were stained with a mouse anti-SWC-3a antibody (CD172a; clone 74-12-15; VMRD, Pullman, WA) and then separated with magnetic microbeads (goat anti-mouse IgG; Miltenyi Biotec). Live/dead assay was examined with propidium iodide (10 µL/200 µL; stock solution: 1 mg/mL; Sigma, Germany). Monocyte-derived dendritic cells were generated in DMEM supplemented with 10% porcine serum (PAN-Biotech, Germany). A total of 1 × 10^6^ cells/mL/well were seeded in 12-well plates and incubated at the porcine body temperature of 39 °C. They were stimulated with IL-4 (50 ng/mL) and GM-CSF (150 ng/mL, both Bioscience) for 7 days and then used for the subsequent in vitro experiments. On day 7, cells were centrifuged (350 × g; RT; 5 min) and detached with a pipette using cold medium. In the next step, cells were centrifuged at 350 × g and seeded for the experiments in DMEM supplemented with 10% porcine serum without IL-4 and GM-CSF.

### Application of substances and target preparation

The obtained DON (D0156; Sigma-Aldrich, Germany) was diluted in acetonitrile (99.6%; Roth, Germany), evaporated with N_2_, and diluted with 0.9% NaCl to a 0.2-mg/mL stock solution. Working dilutions were prepared in cell culture medium. LPS originating from *E. coli* (10 ng/mL; Sigma, Germany) was used.

### Pig experiment

Experiment and procedures were conducted according to the European Community regulations concerning the protection of experimental animals and the guidelines of the German Animal Welfare Act and were approved by the ethical committee of the Lower Saxony State Office for Consumer Protection and Food Safety (file number 33.4-42-502-04-13/1274). In our study, we investigated the effect of DON-contaminated diets combined with intravenous LPS infusion (*Vena jugularis*). The whole study was accomplished using a total of 44 barrows (German Landrace, Mariensee, Germany). The animals had an initial mean body weight (BW) of 25.8 ± 3.7 kg and were divided into 2 feeding groups. The first group was chronically exposed to a diet which contains naturally DON-contaminated maize, and the second group was fed with the same diet but without contaminated maize. Each pig received 6.43 mg DON per day. To investigate the effect of LPS, the left jugular grove was opened to insert a catheter into the vena jugularis externa for application of LPS or 0.9% NaCl in the controls. We examined overall 4 groups: CON_CON (CON-fed/CON-infused), DON_CON (DON-fed/CON-infused), CON_LPS (CON-fed/LPS-infused), and DON_LPS (DON-fed/LPS-infused). Further information about the pig experiment and surgery is found in Bannert et al. (2015).

### Laser capture microdissection (LCM)

Membrane slides (Zeiss; Oberkochen, Germany) were coated with 0.05% polylysine (Sigma; St. Louis, USA) for 30 min, RT, and rinsed with water (Aqua dest., 3 × 5 min; dipping). Furthermore, slides were soaked in “RNAse away spray” (Roth, Karlsruhe, Germany) and washed in DMDC-Aqua dest. (5 min each, RT). Porcine jejunum was embedded in tissue freezing medium (Leica, Wetzlar, Germany). Thin gut sections (10 µm) were prepared in a cryostat at − 18 °C, placed onto membrane glass slides, and dried on a warming plate (2 min; 40 °C). In the next step, thin sections were fixed with − 20 °C pre-cooled 70% ethanol in DMDC-Aqua for 1 min, stained with cresyl violet ((1) 1% cresyl violet solution in 50% ethanol/DMDC-Aqua, 4 °C; 1 min; (2) 70% ethanol-DMDC-Aqua, 2 min, 4 °C; (3) 100% ethanol, 2 min, 4 °C), and dried on a hot plate 40 °C. Areas of interest (lamina propria below the enterocytes in the villi) were marked with a freehand tool (10 × magnification; 3,000,000 µm^2^/sample; Palm Robo 4.4, Zeiss, Oberkochen, Germany). In the next step, using the tool AutoLPC (laser energy cutting: 50%/focus, 83%; LPC, 83%/focus, 81%) areas of interest were removed into adhesive caps (Zeiss, Oberkochen, Germany). Furthermore, 350 µL RLTplus (+ ß-mercaptoethanol) was added to the tube, incubated “up-side-down” for 30 min on ice, and centrifuged for 5 min (RT; 12,000 rpm). Supernatants were added to a “gDNA Eliminator column” (30 s; 8000 × g; Qiagen, Hilden, Germany). Samples were processed after the manufacturer’s instructions (RNeasy Plus Micro Kit, Qiagen, Hilden, Germany). In the last step, RNeasy MinElute spin columns (Qiagen, Hilden. Germany) were eluted with 2 × 14 µL RNase-free water. Quantitative PCR amplification was performed for all genes under the following conditions using a qTower (Analytik Jena, Germany): 10 min at 45 °C for reverse transcription, 2 min at 95 °C (polymerase activation) followed by 40 cycles of 30 s at 95 °C, and 60 s at optimal primer annealing temperature (Table 1). Melting curve analysis (50–95 °C) was used for assessing amplification specificity. The reaction volume of 10 µL contained 5 µL SensiFAST SYBR/No-ROX (2 × , One-Step Mix, Bioline, Germany), 0.4 µL of the respective primers (10 pmol/µL), 2.9 µL nuclease free water, and 1 µL RNA (2 ng/µL). The analysis comprised five animals per group with each sample in triplicates. The ddCt method was used for the calculation of differences in the gene expression (ratio = 2 − ddCt) (Pfaffl 2001). The differences between the samples were normalized to the individual expression of the geometric mean of the two housekeeping genes: ß-actin and 18S (Table 1).

**Table 1:** Used primer sequences

**Name**	**Function**	**Sequence 5′–3′**	**Sequence 3′–5′**	**Temperature (°C)**	**Biorad qTower**	**Efficiency (%)**	**Biorad qTower**
**IL-8**	Interleukin 8	tggcaaattgttaaacgaaca	actgacgttcgaaacaatacg	56.1	55.5	97	101
**GRP78**	Heat shock 70 kDa protein 5	ggggagaaggaattggctatc	aacccagtacacgtagacca	62.4	59.5	96	113
**TLR4**	Toll-like receptor 4	gccaattcaactcttagcgc	tcacctttgacgtagtacca	56.2	56.5	99	111
**CD16**	FcγRIII	ccgaagtctgtggtgatcct	tgtctcacctccaccttacc	61.0	62.5	95	102
**MHCII**	Major human histocompatibility complex class II	gttgaagaacgggcactctg	agagtggaaggagggaagac	61.0	62.5	98	103
**GAPDH**	Glyceraldehyde-3-phosphate-dehydrogenase	acccagaagactgtggatgg	ttccagtagggactcgagtt	56.1	60.5	90	129
**actin**	Cytosceletal protein	tgcactttattgaactggtctca	gtatgaagttcaacgccctgt	60.8	58.5	99	101
**18S**	Ribosomal subunit 18S	gcaattattccccatgaacg	aacctaccaaatcactccgg	57.0	57.5	101	103

### Microarray data processing

MoDCs were seeded in a 12-well culture plate (1 × 10^6^/mL/well) and cultured with DON or LPS (*E. coli*) or with both (CON 0 ng/mL; DON 50 ng/mL; LPS 10 ng/mL; DON 50 ng/mL + 10 ng/mL LPS) for 3 h. TRIzol^®^ reagent (Invitrogen, Germany) was added to samples of MoDCs as described in the manufacturer’s protocol. Briefly, the MoDCs were scrapped from the plate and lysed. Then chloroform was added to the cell suspensions, and RNA was recovered from the aqueous phase by precipitation with isopropyl alcohol. The dried pellet was dissolved in DEPC water (Roche, Germany) and stored at − 80 °C. For holistic expression analysis, 15 samples were selected for subsequent analyses covering all three treatment groups (DON [50 ng/mL], LPS [10 ng/mL], DON/LSP [a combination of both]) and the control group.

For the microarray experiments, individual samples were hybridized on porcine 24 k microarrays according to the manufacturer’s directions (Affymetrix, Santa Clara, CA, USA). The microarray platform covered 24,123 transcript IDs representing 20,689 known genes. The raw data has been deposited in a MIAME compliant database (Edgar et al. 2002), the National Center for Biotechnology Information Gene Expression Omnibus (www.ncbi.nlm.nih.gov/geo) (accession number: GSE250031). Data were processed using R scripts, including the arrayqualitymetrics and the affy packages (Kauffmann et al. 2009; Gautier et al. 2004). The data were GCRMA normalized (Log2). In order to improve statistical power (Bourgon et al. 2010), data were filtered (MAS5) to exclude such probe sets absent in more than 50% of samples within one treatment/control group, respectively. Furthermore, uninformative data were discarded, such as control probe sets and internal controls. Additionally, probe sets with low standard deviations were excluded from further data processing, because the corresponding transcripts would be unlikely to show altered mRNA abundances. Annotation data were obtained as previously described (Naraballobh et al. 2010). The analyses revealed 7819 transcript IDs (corresponding to 5666 genes). Relative mRNA differences were analyzed using a mixed linear model using treatment as fixed effect and day of experimental replicates as random effect (SAS version 9.4; SAS Institute, Cary, NC, USA). To account for multiple testing, *p*-values were converted to a set of *q*-values (Storey and Tibshirani 2003). The level of significance was set at *p* ≤ 0.05 and *q* ≤ 0.05, respectively.

### qPCR

Reverse transcription was performed using the RevertAid First Strand cDNA Synthesis Kit (Fermentas, Germany). Reaction conditions and PCR program followed the manufacturer’s instructions. Different porcine gene–specific primers were used (Table 1). Primer pairs were designed using the Primer3 program (http://frodo.wi.mit.edu/cgi_bin/primer3/primer3_www.cgi.de) based on NCBI porcine gene sequences and purchased by MWG. Real-time PCR amplification was performed as described previously by Nossol et al. (2011). *β-Actin* and *18S* served as housekeeping genes, and the geometric mean of both was calculated for further analysis. Five independent experiments were carried out in sets of three technical replicates (*N* = 5), whose results were averaged and further mathematically processed using the ΔΔ − CT method (Pfaffl 2001).

### Oxygen measurement

The oxygen uptake was measured with a Microx TX3 (Presens, Regensburg, Germany). Microx TX3 is a micro fiber optic oxygen meter with a microsensor based on a 140-µm optical fiber. The sensor was calibrated after the manufacturer’s instructions (manual; 2-point calibration). The oxygen content in the medium (apical from the cells was measured over 10 min. The oxygen uptake is given due to the difference between both values (sample value − blank value _[medium without cells]_)_._

### Lactate and glucose measurement

Supernatants were transferred into tubes, centrifuged (350 × g; 10 min) at room temperature. All samples (24 h, 48 h, and 72 h) were collected and frozen at − 80 °C until measurement. Cell-free cell culture medium was used as blank. Glucose and lactate concentrations were determined immediately using Cobas C 501 (Roche, Germany) as well as reagents of test systems GLUC2 and LACT2 (Roche, Germany), respectively. The differences in glucose and lactate concentration between blank and samples were considered glucose consumption and lactate production. Results are shown as glucose consumption^48h^ [glucose consumption^72h^ − glucose consumption^24h^] and lactate production^48h^ [lactate production^72h^ − lactate production^24h^].

### ATP measurement

Cells were seeded as described above. On day 7, cells were treated with or without DON and/or LPS for 72 h. On day 9, control cells were treated with or without carbonylcyanid-4-trifluormethoxyphenylhydrazon (FCCP, 10 µM, Sigma-Aldrich, Hamburg, Germany) for 24 h. On day 10, cells were centrifuged (350 × g; 5 min; RT), media was removed, and a boiling hot puffer (300 µL/well; 100 mM TRIS; 4 mM EDTA; pH = 7.75, Roth, Karlsruhe, Germany) was added to the cells. In the next step, cells were scraped off the bottom of the well with a cell scraper. The cell suspension was transferred into a tube, incubated for 2 min at 100 °C, and centrifuged at 1000 × g for 60 s. Supernatants were pipetted into 96-well microplate (50 µL/well; triplicates; Greiner bio-one; Frickenhausen, Germany). Samples were kept on ice until measurement. An ATP-standard curve was prepared after the manufacturer’s instructions (5 readings within 5 min; 25 °C; ATP Bioluminescence Assay Kit CLS II; Roche, Basel, Switzerland).

### Western blot analysis

MoDCs were seeded in a 12-well culture plate as described above (1 × 10^6^/mL/well). Western blot analyses were performed as described in Nossol et al. (2023). GRP78 was used as primary antibody (1:1000; Abcam, Germany; blocked with 5% milk) and mouse anti-β-actin as loading control (1:30,000, cell signaling, Germany). The secondary antibody was purchased with the BM Chemiluminescence Western Blotting Kit Mouse/Rabbit (Roche, Germany). Blots were analyzed on an Alpha-Ease^®^ FC Imaging System (Alpha Innotech, Canada). Raw intensities of protein bands were used for statistical analyses. All experiments were carried out three times. The loading control actin was utilized for normalization, and the bands with the highest raw intensity were used to normalize all other actin bands (normalization factor). Subsequently, the normalization factors were applied to the corresponding raw intensities of the investigated protein (GRP78). The normalized control values (CON-group) were used as reference in the boxplot diagrams.

### IL-8 analysis

Levels of IL-8 were measured in culture supernatants using an ELISA kit accordingly to the manufacturer’s protocol (R&D Systems, Germany). Therefore, 1 × 10^6^ cells/mL were seeded in 12-well plates and incubated with DON with/without LPS (*E. coli*) (0 ng/mL DON (CON); 50 ng/mL DON; 10 ng/mL LPS; 50 ng/mL DON + 10 ng/mL LPS). The culture supernatants were collected after each experiment (24 h; 48 h, 72 h), centrifuged at 350 × g, and frozen at − 80 °C. IL-8 was quantified in each supernatant in duplicates following the manufacture’s protocol. Therefore, each sample was diluted (1:1000), and colorimetric results were read on a 96-well plate reader at a wavelength of 450 nm (Tecan, Germany).

### Bioorthogonal non-canonical amino acid tagging (BONCAT)

MoDCs were cultured as described above. On day 7, medium was changed and medium without IL-4 and Gm-CSF was added with the appropriate DON and LPS supplementations. After 24 h, 48 h, or 72 h, medium cells were centrifuged (3 min; 350 × g) and medium was aspirated and changed to medium without AHA and methionine. Cells were incubated for 30 min. In the next step, MoDCs were incubated for up to 2.5 h with medium with appropriate DON and LPS supplementations and AHA. One control for labelling was carried out. After incubation with AHA, cells were centrifuged; medium was aspirated and replaced with a cold medium (wo methionine/wo AHA) to detach the cells from the plate. Furthermore, cells were washed with PBS and stored at − 80 °C.

Tagging of AHA-labelled proteins was performed as essentially described in Dieterich et al. (2007) and Landgraf et al. (2015). Briefly, cell pellets were lysed for 5 min at 95 °C in 300 µL 1 × PBS, pH 7.8, containing 0.2% Triton X100, 0.1% SDS, 1 × cOmplete™ EDTA-free protease inhibitor cocktail (Roche), and 250 U/mL Benzonase^®^ nuclease. The resulting protein extracts were centrifuged for 5 min at 14.000 × g at 4 °C, and the supernatant transferred into fresh 1.5-mL Eppendorf tubes. For click chemistry, samples were supplemented with 0.2 mM Triazole ligand (Tris[(1-benzyl-1*H*-1,2,3-triazol-4-yl)methyl]amine (TBTA), 25 µM biotin-PEO_3_-alkyne-tag, and 0.2 mg/mL copper(I)bromide-suspension (Acros), whereby the samples were thoroughly vortexed for 15 s between each supplementation step. Subsequently samples were incubated under continuous agitation at RT. After a 2-h reaction time, precipitates were removed by centrifugation for 5 min at 3000 × g, 4 °C, and the remaining protein extracts were further processed for quantification, SDS-PAGE, and Western blot.

### Western blot experiments and quantitative analysis

For SDS-PAGE and western blot analysis, protein fractions were solubilized with 4 × SDS sample buffer (250 mM Tris-HCl, pH 6.8, 1% SDS, 40% glycerol, 20% ß-mercaptoethanol, 0.004% bromophenol blue), boiled for 5 min (95 °C), and separated on 5–20% SDS-polyacrylamide gradient gels, subsequently followed by transfer onto nitro cellulose membranes. After blotting, membranes were blocked with a blocking solution (5% dry milk, 0.1% Tween 20 in 1 × TBS) for 1 h. Incubation with primary antibodies (anti-biotin, 1:10.000) was done overnight at 4 °C in blocking solution. After intensive washing, blots were incubated for 90 min at room temperature with HRP-conjugated secondary antibodies (1:10.000) in blocking solution as well and finally developed with ECL reagent (Thermo Fisher Scientific) using the Odyssey^®^ Fc luminescence detector (LI-COR). For normalization, identical samples were separated by SDS-PAGE and stained for 1 h with 0.05% Coomassie brilliant blue dissolved in 50% methanol and 10% acetic acid, followed by distaining using a solution consisting of 5% methanol and 7% acetic acid. Determination and quantification of the western blot signals and Coomassie-stained gels were done using the Image Studio Lite version 5.0 software from LI-COR.

### Acute cytosolic Ca^2+^-response to the application of low and high DON-doses

MoDCs (1 × 10^6^/200 µL) were seeded (2 mL) on glass-bottom dishes (Firma, USA) in DMEM with IL-4 and GM-CSF as described above. All substances for imaging experiments were dissolved in HBSS buffer (Hanks balanced salt solution; Biochrom, Germany). Controls were treated with HBSS alone. Furthermore, all wash steps were performed with HBSS. After cleaning the cells from culture medium (3 times), they were loaded with Fura-2 AM (2 µM, Invitrogen, Germany) in HBSS for 30 min at room temperature in the darkness. Freeing the cells from staining solution by three wash steps was continued by a 15-min post-reaction step in HBSS at 39 °C. Then cells were directly imaged on a Zeiss inverse microscope (Axio Observer.Z1; Zeiss, Germany) equipped with a camera (QImaging Retiga EXI Aqua; TILL Photonics, Germany), a monochromator (Polychrome V, TILL Photonics), and an incubation chamber adjusted to 39 °C (Okolab, Italy). Cellular Ca^2+^-response to DON (0 ng/mL, 10 ng/mL, 50 ng/mL, 200 ng/mL, and 2000 ng/mL) or the positive controls ATP (500 µM) and histamine (100 µM) in HBSS were analyzed after the acute challenge with the substance (frame 60) and a subsequent observation for 20 min. Fluorescence of 340 and 380 nm excitation (*F*_340nm_ and *F*_380nm_) was collected at 510 nm in a frequency of 3 s/frame and an illumination time of 50 ms/frame. Fluorescence ratio (R) was calculated from both wavelengths (*R* = *F*_340nm_/*F*_380nm_). Images were captured with a digital camera by TILLvision software (TILL Photonics) and analyzed by Fiji software (National Institute of Health, USA) on a single-cell level.

### Statistical analysis

All data were tested on normal distribution (Shapiro-Wilk) and variance homogeneity (Levene test). In the case of normal distributed data and variance homogeneity, an ANOVA was performed with a post hoc test (Dunnett, two-sided). In the case of not normal distributed data, multiple tested data were performed with Kruskal-Wallis test and *p*-values were corrected (Bonferroni correction). Results are expressed as means and standard error. Statistical analysis was examined via SPSS 28. Asterisks indicate significant differences (*p ≤ 0.05; **p ≤ 0.01; ***p ≤ 0.001).

## Results

### Isolation and generation of MoDCs

**Fig. 1:**
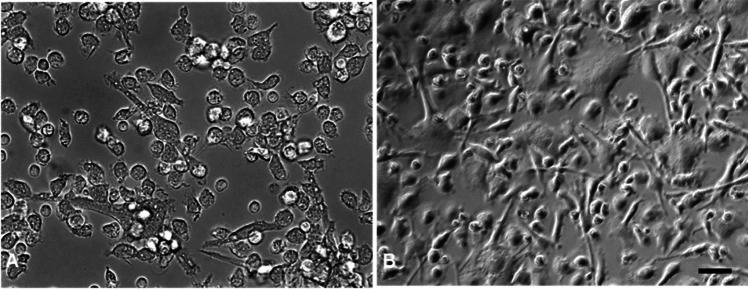
Differentiation of MoDCs in cell culture. The left picture shows MoDCs 24 h after seeding in differentiation media and the right picture shows MoDCs after 6 days of cultivation. A multiplicity of dendrites is visible on the right picture (bar = 10 µm)

MoDCs were successfully separated with a SWC-3a antibody. About 90% of the cells were alive after the procedure. In the next step, cells were cultured with IL-4 and GM-CSF for 7 days. The effect of both additives was checked visually by microscope (Fig. 1). After 1 day of cultivation, only a few cells developed dendrites, but after 3 days, over 70% were positive for dendrites. After 7 days of cultivation, cells were used for the experiments, and all cells showed a dendritic phenotype.

### Effect of DON and/or LPS following a 3-h incubation time on initial gene expression of MoDCs

**Fig. 2:**
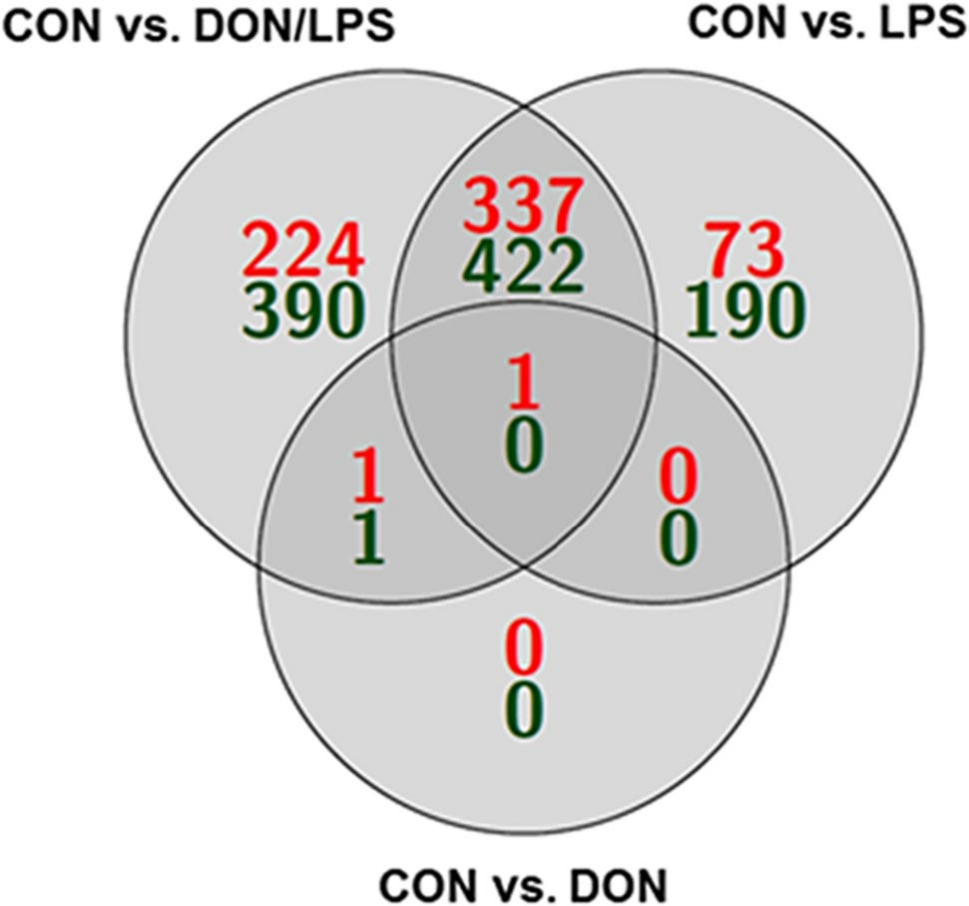
Intersection of DEGs due to DON and LSP exposure in MoDCs. A total of 1639 genes were significantly regulated and used for intersection analysis. In total, 1003 genes were up-regulated (green) and 636 were down-regulated (red). In addition, one gene was significantly down-regulated in all groups: GRP78

In the microarray analyses, 1639 genes were significantly regulated in comparison of CON vs. DON, CON vs. LPS, and CON vs. DON/LPS (Fig. 2). A total of 1003 up-regulated and 636 down-regulated genes were found in the analyses. Subsequently, a functional clustering was performed with DAVID (Database for Annotation, Visualization and Integrated Discovery; https:david.ncifcrf.gov/home) for all 3 comparisons: CON vs. DON (3 genes), CON vs. LPS (1023 genes), and CON vs. DON/LPS (1076 genes). In the comparison of CON vs. DON, only one pathway was identified, which is significantly regulated by DON: protein processing in endoplasmic reticulum (Table 2). CON vs. LPS resulted in over 40 significantly regulated pathways: (1) TNF signaling pathway, (2) necroptosis, and (3) FoxO signaling pathway (for further information, see Table 2). In comparison of CON vs. DON/LPS, we observed also (1) TNF signaling pathway, (2) MAPK signaling pathway, and (3) AGE-RAGE signaling pathway.

**Table 2:** Significant pathways identified in the KEGG database

	**KEGG-pathway**	**Number of genes**	***p*-value**
CON vs. LPS	TNF signaling pathway	22	4.6 × 10^−9^
	Necroptosis	19	9.2 × 10^−5^
	FoxO signaling pathway	17	9.7 × 10^−5^
	MAPK signaling pathway	28	1.1 × 10^−4^
	Lipid and atherosclerosis	22	2.8 × 10^−4^
	IL-17 signaling pathway	13	3.1 × 10^−4^
	Rheumatoid arthritis	13	4.6 × 10^−4^
	NF-kappa B signaling pathway	14	4.9 × 10^−4^
	AGE-RAGE signaling pathway in diabetic complication	13	9.0 × 10^−4^
	Measles	16	1.3 × 10^−3^
CON vs. DON/LPS	TNF signaling pathway	23	2 × 10^−7^
	MAPK signaling pathway	36	2.6 × 10^−5^
	AGE-RAGE signaling pathway in diabetic complication	17	1.4 × 10^−4^
	Proteoglycans in cancer	26	2.5 × 10^−4^
	NF-kappa B signaling pathway	17	3.2 × 10^−4^
	FoxO signaling pathway	19	3.7 × 10^−4^
	HIF-1 signaling pathway		4.0 × 10^−4^
	Amino sugar and nucleotide sugar metabolism	11	4.1 × 10^−4^
	Metabolic pathways	117	4.7 × 10^−4^
	Ferroptosis	10	4.7 × 10^−4^
CON vs. DON	protein processing in endoplasmic reticulum	2	1.9 × 10^−2^

All significantly regulated genes of the corresponding comparison were used to identify significantly regulated pathways in the KEGG database

For further analyses, we examined intersections of genes between the groups (Fig. 2).

A total of 614 genes were found to be regulated “uniquely” in the comparison of CON vs. DON/LPS. These genes are involved in different pathways like (1) metabolic pathways, (2) proteoglycans in cancer, (3) amino sugar and nucleotide sugar metabolism, (4) glycosaminoglycan metabolism, and (5) HIF-1 signaling pathway. The comparison of CON and DON showed no “uniquely” genes in the DON group. Furthermore, CON vs. LPS resulted in 263 “uniquely” genes, which can be related to different pathways: (1) osteoclast differentiation, (2) PPAR signaling pathway, (3) FoxO signaling pathway, and (4) fatty acid metabolism. With the focus on DON treatment, we found only 3 regulated genes: MAPK1IP1L (mitogen-activated protein kinase 1 interacting protein 1 like), GRP78 (HSPA 5), and DNAJA1 (DnaJ heat shock protein family (Hsp40) member A1). GRP78 was also found in the comparison of CON vs. LPS and CON vs. DON/LPS.

### Comparison of RNA expression in vivo and in vitro

Different genes, which are involved in the KEGG pathways, were analyzed via qPCR. We used the material of the lamina propria mucosae of jejunal gut segments of treated pigs for our qPCR analyses. And we compared it with long-term–treated (24 h and 72 h) porcine MoDCs (Table 3). We observed no significant differences with the focus on IL-8 in the lamina propria mucosae of the treated pigs, but significantly increased values in the LPS-treated (293.49%; *p* < 0.05) and DON/LPS-treated MoDCs (415.06%; *p* < 0.01) compared to the CON group after 24 h. In contrast, just a trend of up-regulation of IL-8 was found in the treatment groups (LPS *p* = 0.061; DON/LPS *p* = 0.083). GRP78 showed no regulation after 24 h or 72 h treatment of MoDCs. In the lamina propria mucosae, a significantly higher GRP78 expression was detected in the CON_LPS (285.67%; *p* < 0.001)-treated pigs compared to the control group (= 100%). No differences were observed in the expression of TLR4, CD16, and MHCII independent of treatment in MoDCs as well as in pigs. Furthermore, we analyzed the expression of GAPDH and found a trend of a higher expression in the DON_LPS-treated (246.52%; *p* = 0.072) pigs compared to the control group.

**Table 3:** qPCR data of treated MoDCs (left) and laser dissection (right, lamina propria mucosae) PCR data [relative quantification; %] of different genes are shown in the table (N = 5). All treatment groups were compared to control [=100%] (*p* < 0.05 *; *p* < 0.001 ***)

**Gene**		**MoDCs**				**Lamina propria mucosae**			
		**CON**	**DON**	**LPS**	**DON/LPS**	**CON_CON**	**DON_CON**	**CON_LPS**	**DON_LPS**
**IL-8**	24 h	100	187.47	293.49*	415.06**	100	61.65	89.16	94.67
	72 h	100	108.67	179.83^T^	185.75^T^				
**GRP78**	24 h	100	94.17	92.44	85.07	100	123.69	285.67**	246.90
	72 h	100	106.19	94.17	92.87				
**TLR4**	24 h	100	122.55	123.68	117.55	100	186.00	87.01	86.60
	72 h	100	123.68	123.11	120.86				
**CD16**	24 h	100	133.48	128.34	155.83	100	96.56	72.09	65.51
	72 h	100	149.48	126.58	156.55				
**MHCII**	24 h	100	122.55	86.65	95.93	100	90.40	110.36	91.60
	72 h	100	128.34	130.13	107.67				
**GAPDH**	24 h	100	131.65	123.97	135.97	100	348.07	183.33	246.52^T^
	72 h	100	109.68	107.18	98.62				

### GRP78

**Fig. 3:**
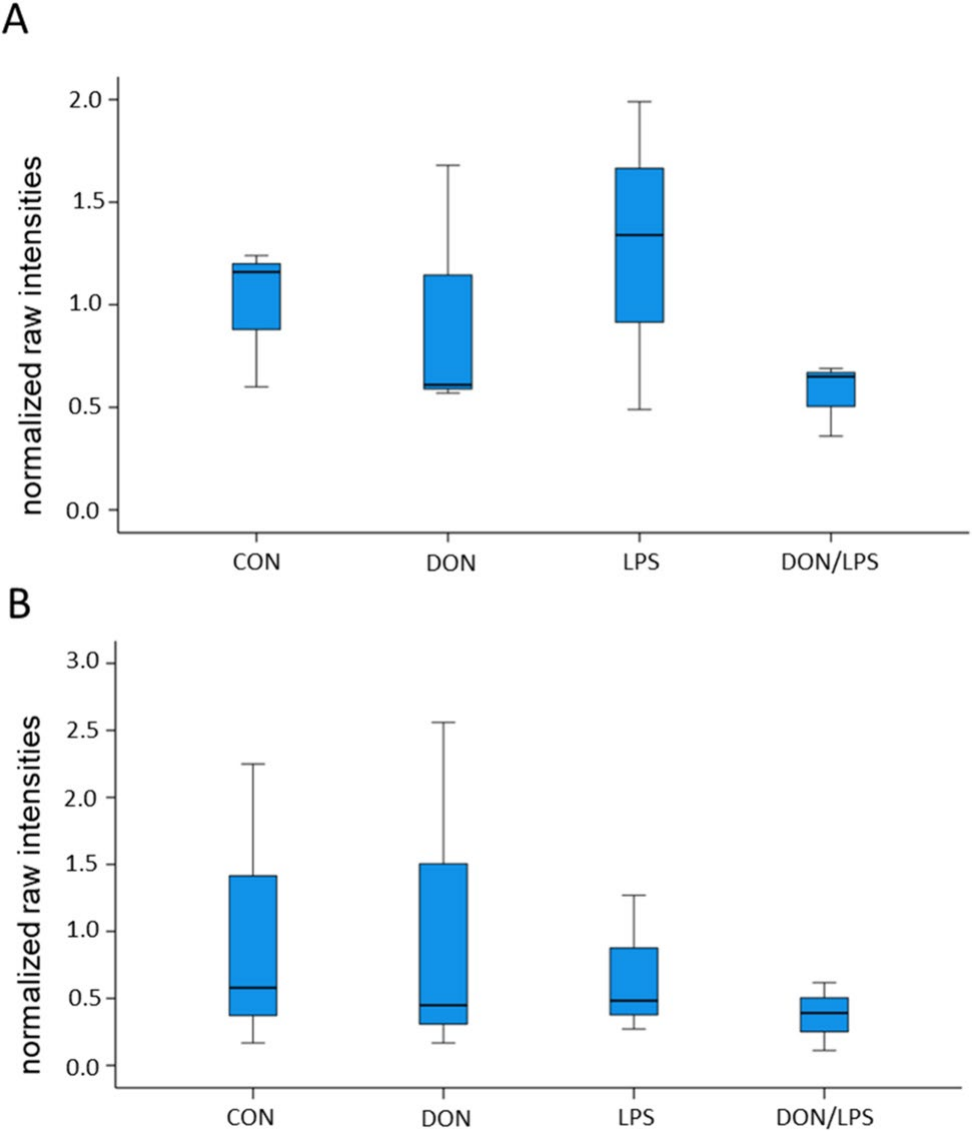
Western blot analyses of GRP78 in primary porcine MoDCs. **A** After 48 h treatment, we detected no significantly different expression of GRP78 in the treatment groups, but numerical lowered content in the DON/LPS group. **B** We observed no differences after 72 h incubation in the treatment groups

GRP78 was analyzed with western blot analyses (Fig. 3) after 48 h and 72 h of incubation with DON (50 ng/mL), LPS (10 ng/mL), and DON/LPS. No significant differences were found in the protein expression of GRP78 after 48 h and 72 h, but the lowest amount of GRP78 was observed in the DON/LPS group independent of incubation time.

### IL-8 production

**Fig. 4:**
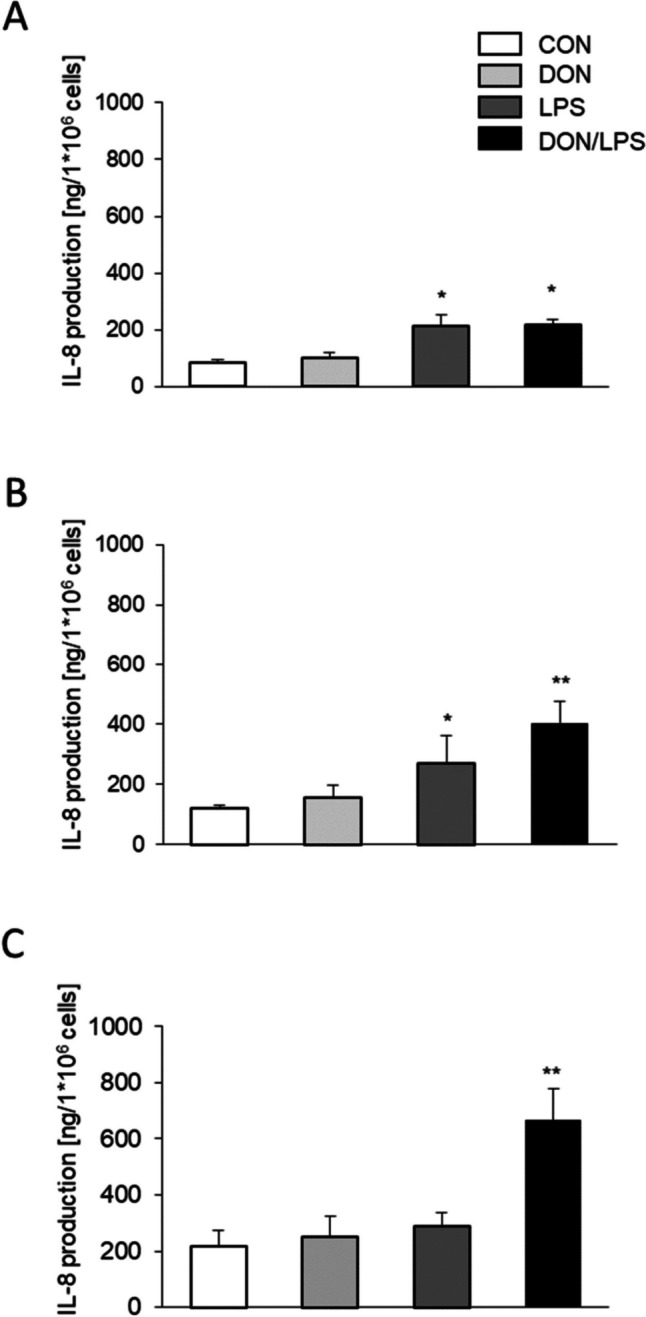
IL-8 production of MoDCs. MoDCs were treated for **A** 24 h, **B** 48 h, or **C** 72 h with DON 50 ng/mL, LPS 10 ng/mL, or DON 50 ng/mL + LPS 10 ng/mL

After 24 h, a significant increase in the IL-8 production was observed in the LPS (215 ng/mL/1 × 10^6^ cells) and DON/LPS group vs. control group (CON, 83.33 ng/mL/1 × 10^6^ cells; both, *p* = 0.013; Fig. 4). Significantly increased IL-8 production was found in the comparison of CON vs. LPS (CON 120.95 ± 10.72 ng IL-8/1 × 10^6^ cells; LPS 268.57 ± 92.90 ng IL-8/1 × 10^6^ cells; *p < 0.05) and CON vs. DON/LPS (402.62 ± 75.08 ng IL-8/1 × 10^6^ cells; **p < 0.01) after 48 h of incubation. Furthermore, a synergistic effect of DON and LPS was observed after 72 h (DON/LPS, 667.14 ± 110.34 ng IL-8/1 × 10^6^ cells; **p < 0.01).

### Oxygen consumption under DON and LPS treatment

**Fig. 5:**
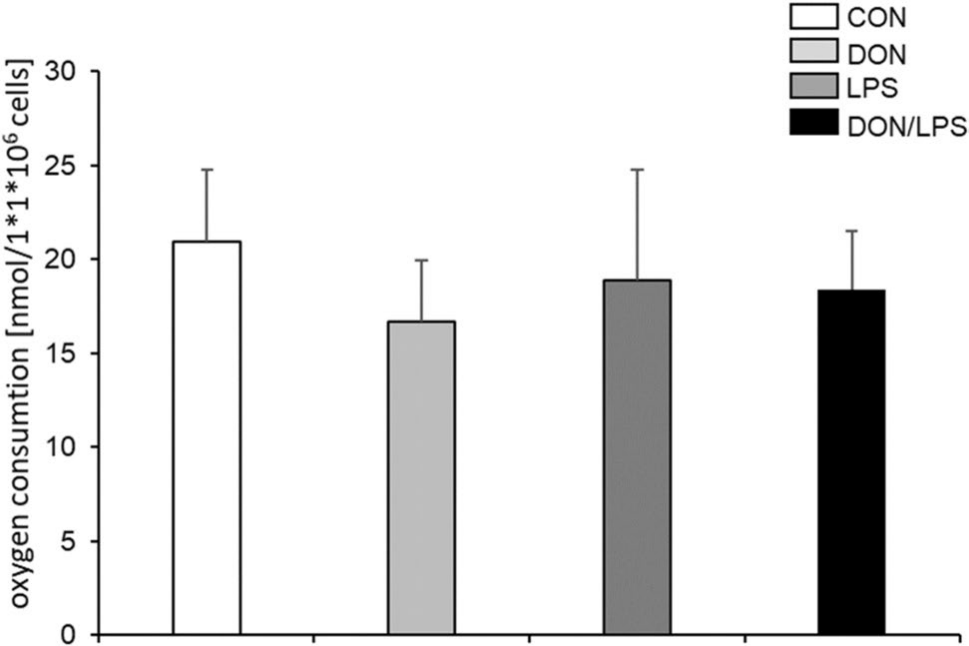
Oxygen consumption (*N* = 5). After 72 h of incubation, oxygen consumption was examined, which resulted in no significant differences between the treatment groups and control group. CON group showed the highest values 20.93 nmol/1 × 10^6^ cells and DON group in the lowest of 16.67 nmol/1 × 10^6^ cells

In the next step, we analyzed oxygen consumption under DON and/or LPS treatment at 72 h (Fig. 5). We found no significantly differences between treatment groups compared to control. The control group showed the highest consumption of 20.93 nmol/1 × 10^6^ cells closely followed by 18.0 nmol/1 × 10^6^ cells (LPS) and 18.41 nmol/1 × 10^6^ cells (DON/LPS). The nominal lowest oxygen consumption showed the DON treatment group with 16.67 nmol/1 × 10^6^ cells.

### Glucose and lactate content

**Fig. 6:**
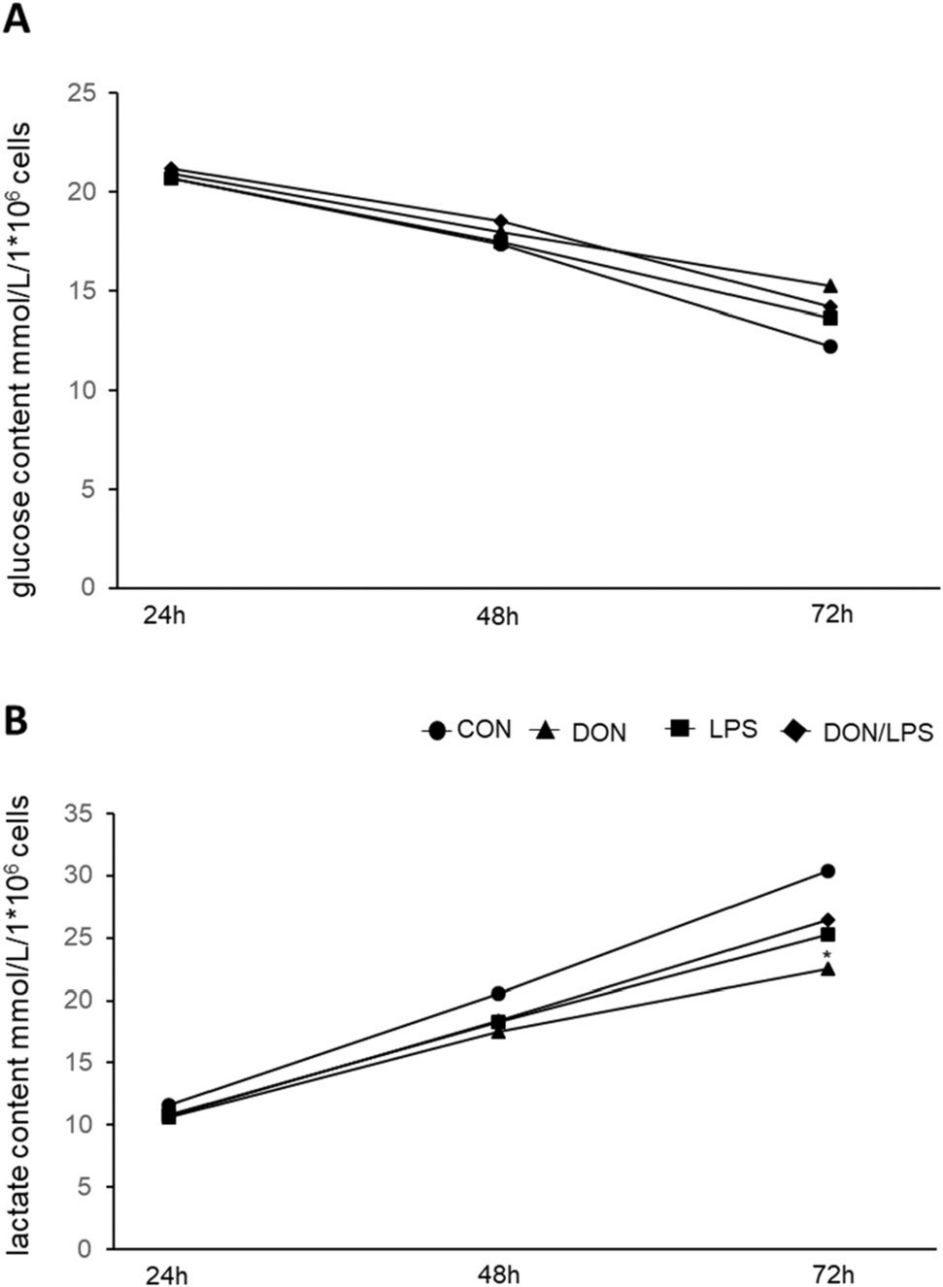
Lactate production and glucose content (*N* = 3). **A** Glucose content of the medium was analyzed. After 24 h of incubation time, similar values for glucose content were found. After 72 h, CON group showed the lowest value for glucose content (12.20 ± 1.45 mmol/L/1 × 10^6^ cells) and DON group the highest (15.27 ± 0.69 mmol/L/1 × 10^6^ cells). **B** After 72 h incubation time, significantly lower lactate values were found in the DON group (22.29 ± 0.95 mmol/L/1 × 10^6^ cells) compared to CON-group (30.34 ± 3.1 mmol/L/1 × 10^6^ cells)

In Fig. 6, lactate content and glucose content after 24 h, 48 h, and 72 h incubation time are shown. At 24 h of incubation, similar values of glucose content were found for all treatment groups: CON 20.67 ± 0.49 mmol/L/1 × 10^6^ cells, DON 20.97 ± 0.55 mmol/L/1 × 10^6^ cells, LPS 20.70 ± 0.76 mmol/L/1 × 10^6^ cells, and DON/LPS 21.17 ± 0.67 mmol/L/1 × 10^6^ cells (Fig. 6A). After 72 h, we observed lower values in all treatment groups compared to 24 h: CON 12.20 ± 1.45 mmol/L/1 × 10^6^ cells, DON 15.27 ± 0.69 mmol/L/1 × 10^6^ cells, LPS 13.63 ± 1.18 mmol/L/1 × 10^6^ cells and DON/LPS 14.20 ± 0.7 mmol/L/1 × 10^6^ cells, but analysis resulted in no significant differences between the groups at 72 h.

In contrast, lactate production had a significantly faster increase in CON group 24 h, 11.65 ± 1.1 mmol/L/1 × 10^6^ cells to 72 h, 30.34 ± 3.1 mmol/L/1 × 10^6^ cells over time compared to the DON group 10.64 0.64 mmol/L/1 × 10^6^ cells to 72 h, 22.49 ± 0.95 mmol/L/1 × 10^6^ cells (Fig. 6B; *p* = 0.029).

### ATP content

**Fig. 7:**
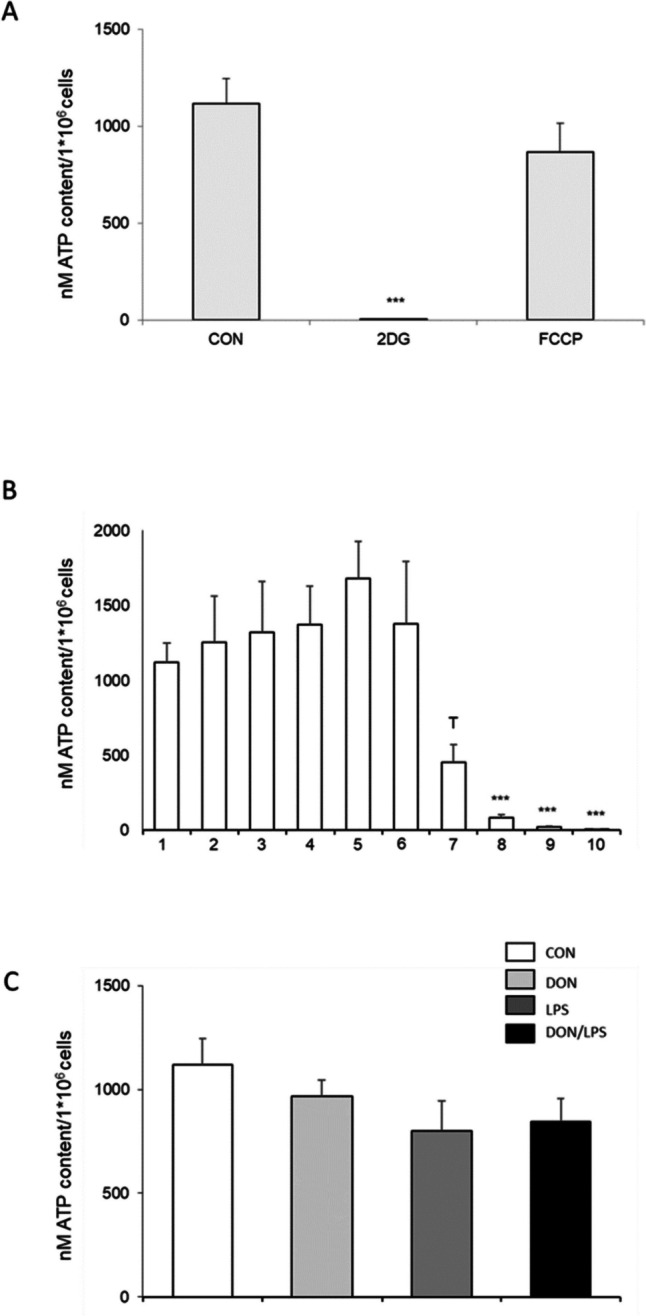
ATP content (*N* = 3). **A** MoDCs were treated with 2DG or with FCCP. The incubation of 2DG resulted in a significant decrease in ATP content (1.03 nM/1 × 10^6^ cells; ***p < 0.001) but no difference was found with FCCP (864.99 nM/1 × 10^6^) compared to control (1117.07 nM/1 × 10^6^ cells). **B** Different DON concentrations were used to analyze the effect of DON on ATP content. A significant decrease was observed with DON 400 ng/mL up to 1000 ng/mL (400 ng/mL, 80.99 nM/1 × 10^6^; 500 ng/mL, 20.49 nM/1 × 10^6^; 1000 ng/mL, 2.55 nM/1 × 10^6^; all, ***p < 0.01). The concentration of 300 ng/mL resulted in a trend of a lower ATP content (454.41 nM/1 × 10^6^; ^T^*p* = 0.078) compared to CON. **C** With the focus on the different treatment groups, DON, LPS, and DON/LPS, we observed no significant differences between control and treatment groups but a marked decrease was found in the LPS group (801.06 nM/1 × 10^6^ cells) compared to control

MoDCs were seeded as described above. 2-Desoxy-deglucose and FCCP were used to analyze the metabolic state of the MoDCs (Fig. 7A). A significantly decreased ATP-content was found with 2DG (1.03 nM/1 × 10^6^ cells; *p* < 0.001) Furthermore, a treatment with FCCP (864.99 nM/1 × 10^6^) showed no significant decrease in ATP in comparison to control (1117.07 nM/1 × 10^6^ cells; Fig. 7A). To examine the effect of DON on ATP content, different concentrations were used for this experiment (Fig. 7B). A significant decrease was observed with DON 400 ng/mL up to 1000 ng/mL (400 ng/mL, 80.99 nM/1 × 10^6^; 500 ng/mL, 20.49 nM/1 × 10^6^; 1000 ng/mL, 2.55 nM/1 × 10^6^; all, *p* < 0.01). The concentration of 300 ng/mL resulted in a trend of a lower ATP content (454.41 nM/1 × 10^6^; *p* = 0.078) compared to control (1117.07 nM/1 × 10^6^ cells). With the focus on the different treatment groups, we observed no significant differences between control and treatment groups, but a marked nominal decrease was found in the LPS group (801.06 nM/1 × 10^6^ cells; Fig. 7C) compared to control.

### Analyses of the protein biosynthesis

**Fig. 8:**
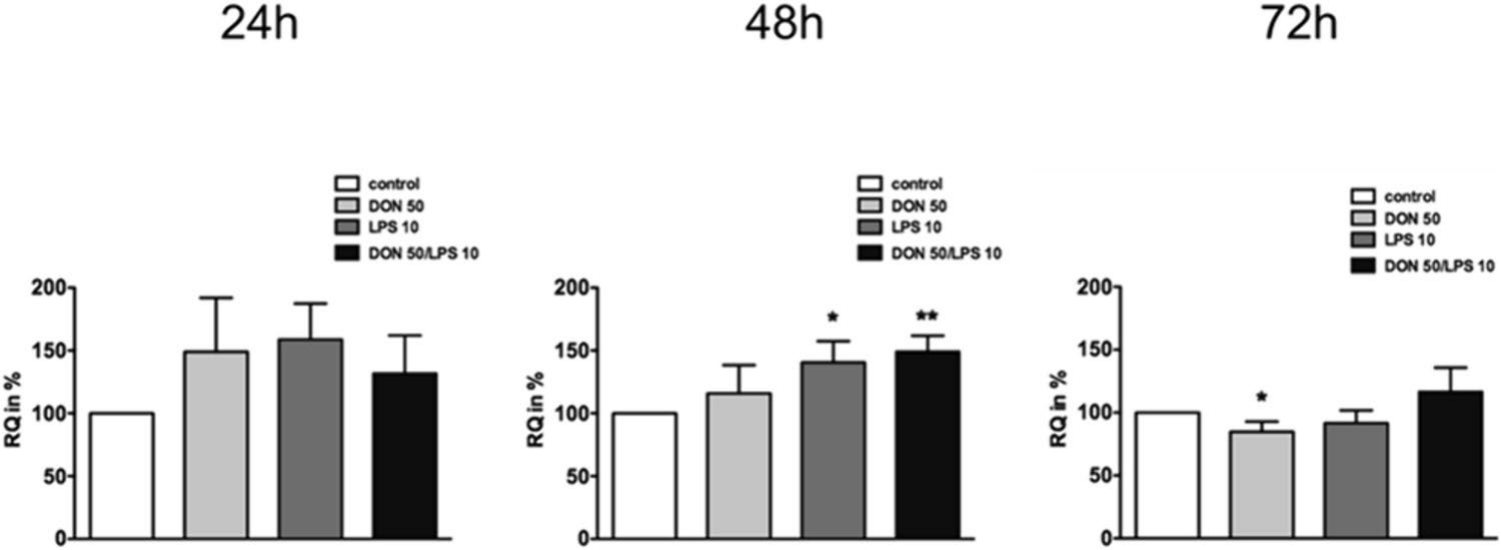
Analysis of protein biosynthesis in MoDCs (*N* = 3). No significant differences in protein biosynthesis were observed after 24 h incubation with DON 50 ng/mL, LPS 10 ng/mL, or both DON 50 ng/mL and LPS 10 ng/mL. After 48 h, the treatment groups LPS 10 ng/mL (*p < 0.05) and DON 50 ng/mL + LPS 10 ng/mL (**p < 0.01) resulted in a significantly higher protein biosynthesis compared to control group (CON). Additionally, a significantly decreased protein biosynthesis was found in the DON treatment group after 72 h (*p < 0.05)

New synthesized proteins were analyzed through the use of AHA (Fig. 8). Therefore, cells were pre-incubated with DON, LPS, and DON/LPS for 24 h, 48 h, and 72 h. After 24 h, a marked increase in new synthesized proteins was observed in DON, LPS, and DON/LPS. After 48 h, a significant increase in the LPS and DON/LPS group in comparison to control was detected. Furthermore, after 72 h, a significant decrease in new synthesized protein was found in DON group (DON, *p* ≤ 0.05*).

### Ca^2+^ response to acute DON application

**Fig. 9:**
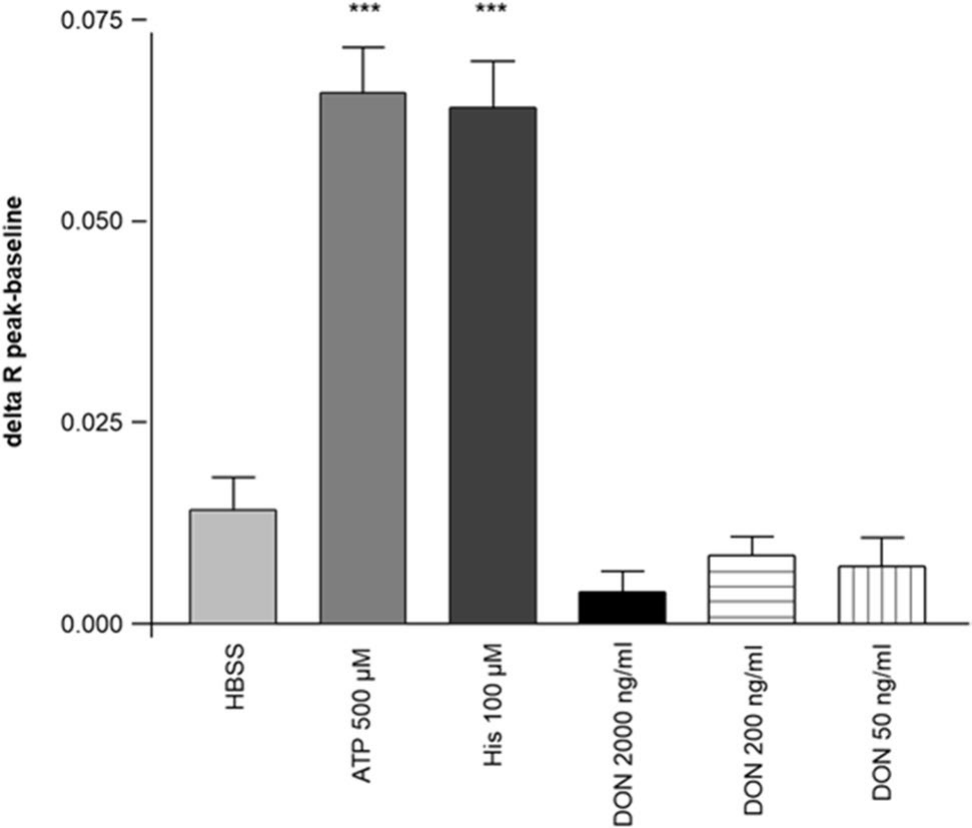
Ca^2+^-response to acute DON application. The experiment was repeated 5 times with not less than 20 cells in each experiment. Three different DON concentrations were used: 50 ng/mL, 200 ng/mL, and 2000 ng/mL for calcium imaging with Fura. We detected no Ca^2+^-response with all three different concentrations. HBSS was used as negative control and 500 µM ATP but also 100 µM histamine was used as positive control. Both positive controls trigger a calcium response in MoDCs (***p < 0.001)

Three different DON concentrations were examined: 50 ng/mL, 200 ng/mL, and 2000 ng/mL, but no calcium-response was observed (Fig. 9). To check these results, two positive controls were carried out. Due to the application of ATP as well as histamine, a significantly increased calcium response was observed. In the last step, a control with HBBS was realized and no calcium response as observed in the DON-treated MoDCs was detected.

## Discussion

Deoxynivalenol can stimulate or suppress immune function and it depends on dose and duration of exposure (Pestka et al. 2004). Low doses of DON evoke expression of a variety of immune- and inflammation-related genes like IL-6 (Sugita-Konishi and Pestka 2001; Wong et al. 2001), IL-2 (Li et al. 1997), TNFα (Chung et al. 2003), and COX-2 (Moon et al. 2003). On the other side, DON is known to increase the binding of several transcription factors like NF-kB (Ouyang et al. 1996) and AP-1 (Li et al. 2000). In our study, we analyzed the effect of the low dose of DON (50 ng/mL) with or without a low concentration of LPS (10 ng/mL) on primary porcine monocyte–derived dendritic cells on the initial response on RNA level (3 h after treatment) with a microarray analysis. The low concentration of 50 ng/mL DON was also found in the peripheral blood of pigs (Danicke et al. 2004). LPS, which is a glycan-based gram-negative pathogen-associated molecular pattern (PAMP), induces the production of various proinflammatory mediators to control bacterial infection. In our microarray analyses, we found an activation of the TNF signaling pathway even after 3 h of incubation with LPS 10 ng/mL and with DON 50 ng/mL + LPS 10 ng/mL. Schwarz et al. demonstrated that 0.2 ng/mL LPS could induce the production of TNF-α and IL-6 by CD14^+^ monocytes. In contrast, Chaiwut and Kasinrerk (2022) showed that 0.0025 ng/mL and 0.005 ng/mL LPS, respectively, can induce TNF-α and IL-6 production in human primary monocytes (Chaiwut and Kasinrerk 2022). Chung and co-workers observed an activation of TNF-α after 2 h of incubation with 100 ng/mL LPS and in the combination of DON 100 ng/mL + 100 ng/mL LPS (Chung et al. 2003). We used in our study a tenth of LPS concentration. In our study, a significantly increased IL-8 RNA expression was found in 10 ng/mL-stimulated LPS MoDCs and a synergistic effect in LPS and DON/LPS-treated MoDCs after 24 h. After 72 h, only a trend of up-regulation was found in both groups. In contrast, a significant higher IL-8 production was observed in LPS and DON/LPS-stimulated MoDCs after 24 h and 48 h, but a synergistic effect of both was only detected after 72 h of incubation. The RNA expression level of CON-/DON-fed and LPS-infused pigs respectively showed no differences between the groups. The induction of IL-8 due to DON application was also shown in PBMCs (Islam et al. 2006). Islam and co-workers showed that a concentration of 250 ng/mL up to 1000 ng/mL was necessary to induce IL-8 production. IL-8 is a well-known pro-inflammatory cytokine inducer and is involved in the pathogen-induced alterations of the tight junctions (Otte and Podolsky 2004). Roselli and co-worker showed that the disruption of the membrane barrier due to ETEC was associated with a strong increase of IL-8 secretion. After blocking IL-8 activity, the damages disappeared (Roselli et al. 2007). Our results show that the stimulation of dendritic cells with low DON dose and LPS dose significantly increases the IL-8 release. Additionally, also LPS during infection also activates IL-8 expression in enterocytes (Angrisano et al. 2010). Therefore, we hypothesize that the high IL-8 release disrupts the intestinal barrier, the LPs concentration will increase, and consequently, IL-8 release will increase in MoDCs because it is regulated in a dose-dependent manner (Hu et al. 2014). In the second step, LPS enter the barrier and additionally increase the IL-8 secretion which increases the disruption of the barrier.

Maresca and co-worker demonstrated the activation of NF-кB pathway is involved in the increased IL-8 secretion due to DON treatment in human intestinal epithelial cells (Maresca et al. 2008), and Gray and Pestka examined also the involvement of NF-kB in the induction of IL-8 production in human monocytes (Gray and Pestka 2007). In their study, they found that the IL-8 expression is highly NF-kB dependent. P65, p50, and 52 are the three subunits of NF-kB, whereas p65 is the only subunit which contains a transcriptional domain (Gray and Pestka 2007). In our microarray analyses, we observed also an activation of the NF-kB signaling pathway in both treatment groups: LPS and DON/LPS after 3 h exposure. A key function of NF-kB is controlling the immune response at various stages and can be activated through different stimuli, for example, Ca^2+^-release out of the ER-calcium stores. We demonstrated that no calcium signal was observed as response to acute DON application at different concentration. We suggested that DON does not act as a ligand for a receptor, which provide the IP3 signaling pathway (Taylor and Thorn 2001).

Dendritic cells recognize bacteria and viruses through pathogen-associated molecular patterns (PAMPS) (Janeway and Medzhitov 2002). The Toll-like receptor (TLR) family is among these receptors. In our analyses, we detected a higher but not significantly increased TLR4-RNA-expression in all treated MoDCs-groups. TLR4 is also involved in the induction of Nf-kB protein after recognition of bacterial lipopolysaccharide (LPS). Furthermore, the DON-fed (186%) pigs showed also an up-regulation of TLR4-RNA in the lamina propria compared to the control (100%). Recent studies indicate that the LPS-induced MyD88–dependent signaling pathway triggers and also modulates the cell metabolism. Here, TRAF6 interacts with TBK1. TBK1 in turn activates Akt kinase and leads to a rapid enhancement of the glycolysis (Everts et al. 2014; Tan and Kagan 2019). The LPS-stimulated glycolysis and subsequent synthesis of acetyl CoA can be used for expansion of the endoplasmic reticulum and Golgi apparatus which are necessary for intense production and secretion of cytokines. The treatment with DON/LPS resulted also in significantly changes in the metabolic pathways which we could show in our microarray analysis. Therefore, we examined the glucose and lactate content of the treated MoDCs after 24 h, 48 h and 72 h. With the focus on glucose, we detected no differences between the groups, but DON-treated MoDCs released significantly less lactate after 72 h compared to the control group. Our isolated primary MoDCs seem to prefer glycolysis as main metabolic pathway, which could be examined due to the measurement of ATP content in the cells after blocking with FCCP or 2DG. The blocking of FCCP resulted in no significantly decreased ATP content of the cells, but with 2DG, a significant decrease was observed. FCCP is an uncoupler of the oxidative phosphorylation (Heytler 1979) and has a protonophore effect that will dissipate the proton gradient, thereby reducing the ATP synthesis. Oxygen consumption is closely related to ATP. We analyzed oxygen consumption with a fiber electrode. We found no significant differences between our treatment groups but also a slight decrease. The same was found in ATP content with the focus on the treatment groups: DON, LPS, DPN/LPS. In contrast, we detected a significantly decrease in ATP content in MoDCs which were treated with DON in a dose-dependent manner. A trend of a decrease was found in the treatment group of 300 ng/mL but lower concentrations showed no significant decrease. Significantly lower ATP content is also found in patients with SIRS (Singer 2014). Singer and co-workers demonstrated that skeletal muscle ATP concentrations were approximately two times lower in patients with sepsis who subsequently died compared to septic patients who survived and to the control group (Singer 2014). On the other hand, ATP is important in heart and vascular function (Erlinge and Burnstock 2008), during pregnancy (Spaans et al. 2014), and in immune responses (Junger 2011). Under physiological conditions, very low concentrations of extracellular ATP (400–1000 nM) (Bakker et al. 2007; Ryan et al. 1996) and adenosine (40–80 nM) (Ontyd and Schrader 1984) are found. Under necrosis or apoptosis (Gallucci and Matzinger 2001), hypoxia, or inflammation (Bours et al. 2006), ATP is released from cells into the extracellular space. Extracellular concentration of ATP is 3 or more fold higher (Bodin and Burnstock 1998). Extracellularly, ATP acts as danger-associated molecular patterns (Bours et al. 2006; Jacob et al. 2013). Low concentration of DON and/or low LPS concentration showed a slight decrease in ATP. Further studies have to be done with the focus on long-term exposure of low concentrations.

As mentioned above, LPS-stimulated glycolysis and subsequent synthesis of acetyl-CoA can be used for the expansion of the endoplasmic reticulum and Golgi apparatus which is necessary for intense production and secretion of cytokines and chemokines. Not surprisingly, we observed a significantly higher de novo protein expression in LPS and DON/LPS-stimulated MoDCs after 48 h of treatment. This effect disappeared after 72 h, but DON inhibits de novo protein biosynthesis after 72 h. An indication of a disturbed protein biosynthesis/processing was observed in the microarray analysis. Here, the “protein processing in the endoplasmatic reticulum” was given as the exclusively regulated pathway. Different studies indicate an effect of DON on the protein expression through a DON-induced translation inhibition (Pan et al. 2014). In this context, GRP78 has an essential role. GRP78 is essential for normal function of ER (Angrisano et al. 2010) and is also involved in many cellular processes, including the newly synthesized polypeptides across the ER membrane, facilitating the folding and assembly of proteins. GRP78 is significantly decreased in all 3 treatment groups, and we detected no differences in the western blot analyses between the groups. On the other hand, GRP78 is the master regulator of ER stress, and this causes Ca^2+^ release out of ER-calcium stores. Mitochondria and ER are interconnected both physically and physiologically, affecting mitochondrial metabolism but also complex cellular processes (Rizzuto et al. 1998). Mitochondria modulate and synchronize Ca^2+^ signaling. Special stimuli, which generate IP_3,_ cause Ca^2+^ release from the ER, and Ca^2+^ is rapidly taken up. This can induce apoptotic pathways (Clapham 2007). In our study, we did not detect any calcium response on acute DON application with high or low DON concentration. In a recent study, the effect of DON-contaminated feed and a subsequent LPS challenge on the Ca^2+^ retention capacity (CRC) of porcine liver mitochondria was analyzed, but no significant differences were found between the treatment groups (Renner et al. 2017).

## Conclusion

Our study indicates a synergistic effect of the combined application of practical-relevant DON concentration and LPS on IL-8 in primary MoDCs, which goes along with a significantly increased de novo protein biosynthesis after 48 h of incubation with LPS or with DON/LPS. We postulate that the up-regulated protein expression can be explained by the expansion of the endoplasmic reticulum and Golgi apparatus which are necessary for intense production and secretion of cytokines in stimulated MoDCs. Furthermore, deoxynivalenol leads to a reduced de novo protein biosynthesis after 72 h of incubation, which confirms prior studies, in which is postulated that DON inhibit protein biosynthesis. We hypothesize, that ER stress due to misfolded proteins can inhibit protein biosynthesis. Perhaps, a Ca^2+^ response is observed later but not in the acute application of DON.

## Data Availability

The raw data has been deposited in the National Center for Biotechnology Information Gene Expression Omnibus (www.ncbi.nlm.nih.gov/geo; accession number: GSE250031).
